# Antimicrobial action of methanolic seed extracts of *Syzygium cumini* Linn. on *Bacillus subtilis*

**DOI:** 10.1186/s13568-017-0500-4

**Published:** 2017-11-02

**Authors:** Alok Kumar Yadav, Saurabh Saraswat, Preeti Sirohi, Manjoo Rani, Sameer Srivastava, Manish Pratap Singh, Nand K. Singh

**Affiliations:** Department of Biotechnology, Motilal Nehru National Institute of Technology, Allahabad, UP 211004 India

**Keywords:** *Syzygium cumini*, Antimicrobial, Inhibition, *Bacillus subtilis*

## Abstract

Phytochemicals of *Syzygium cumini* are used for the treatment of various diseases as a traditional medicine but the mechanism behind their action is not well reported. Antimicrobial activity of methanolic seed extract of *S. cumini* was done by agar well diffusion assay on *Bacillus subtilis* and its zone of inhibition was found to be 20.06 mm in comparison to control having no zone of inhibition. MIC of *S. cumini* was found to be 0.3 mg/ml. Genomic DNA degradation of *B. subtilis* reveals apoptosis and FE-scanning electron microscope indicates cell wall cracking on several intervals of time. Results of propidium iodide staining showed few bacterial cells were stained in control; however population of stained cells increased after exposing them for varying period of time. Flow cytometric kinetic data analysis on the membrane permeabilization in bacterial cell showed the significant contribution of antimicrobial potential of the seed extract on antimicrobial-induced permeabilization. In silico analysis revealed two components of *S. cumini* methanolic extract to be active against four enzymes (PDB ID—1W5D, 4OX3, 3MFD and 5E2F) which are crucial for plasma membrane synthesis in *B. subtilis*. Moreover lupeol showed highest binding energy for macromolecule 1W5D and 4OX3 forming one hydrogen bond each whereas stigmasterol showed the highest binding energy for macromolecule 3MFD and 5E2F forming four hydrogen bonds and alkyl bonds respectively. It demonstrates that methanolic seed extracts of *S. cumini* could be used for inhibition of food born infection caused by *B. subtilis* and also an alternative of prevalent antibiotics.

## Introduction

Antibiotic resistance in microorganisms is the major problem in all over the world both in developing as well as developed nations. The antibiotic resistance in bacteria has made it difficult to control some food born infections. The emergence of antimicrobial resistance in bacteria against certain antibiotics has challenged researchers to find some alternatives for avoiding this problem. *Syzygium cumini* are rich in herbal active constituents which have many antimicrobial compounds and could be suggested as an alternative to this problem. *Syzygium cumini* is widely used medicinal plant in herbal medicines due to its vaunted property. Its biological efficiency are noticed as antihyperglycemic, anti-inflammatory, antibacterial, cardioprotective and antioxidant (Kumar et al. 2008; Rekha et al. 2008; Sharma et al. 2008; Tanwar et al. 2011; Mastan et al. 2009; Arun et al. 2011; Tanwar et al. 2011). The antimicrobial activity of *S. cumini* seed extract is effective against *Bacillus subtilis* as these are reported cause food borne illness (Kramer and Gilbert 1989; Drobniewski 1993). The *B. subtilis* strains are found responsible for ropiness in spoiled bread dough (Kirschner and von Holy 1989; Collins et al. 1991). While some of them are also found to be causative agent for septicaemia in immunocompromised patients (Oggioni et al. 1998). Retrospective studies have shown that *B. subtilis* has evolved antibiotic resistance and produces heat stable toxins amylosins (Richard et al. 1988; Apetroaie-Constantin et al. 2009). Furthermore *B. subtilis*, *B. anthracis* and *B. thuringiensis* have spores that protect them from various adverse conditions and found that molecular iodine is quite effective in killing of its spore (Li et al. 2016). In present article the underlying mechanism behind the antimicrobial potential of methanolic seed extract of *S. cumini* on *B. subtilis* is revealed.

## Materials and methods

### Collection of *S. cumini* fruits and extract preparations

*Syzygium cumini* seeds were obtained from orchard of Motilal Nehru National Institute of Technology, Allahabad. Collected seeds were washed in distilled water for about 15 min. Washed seeds were dried completely in an oven at 60 °C for 2–3 days to remove water content and grinded into fine powder. The dried fifteen grams powder of *S. cumini* seed was filled into the thimble and subjected to soxhlet extraction using methanol as solvent and isolated seed extract was concentrated by rotary evaporator. Concentrated extract was dissolved in PBS making a final concentration of 25 mg/ml for use in further study.

### Culture of *B. subtilis* MTCC 2413

*Bacillus subtilis* MTCC 2413 strain was obtained from Microbial Type Culture Collection (MTCC), Institute of Microbial Technology, Chandigarh, India. The culture of *B. subtilis* 2413 was grown in LB broth after transferring few amount of lyophilized powder in it. The broth was kept at 37 °C inside shaking incubator for overnight at 120 rpm as per the recommendation. The overnight culture of *B. subtilis* MTCC 2413 (CFU 10^7^/ml) was used for subsequent study.

### Antibacterial assays

The antibacterial activity of *S. cumini* Linn. was done by using agar well diffusion assay (Valgas et al. 2007). The *B. subtilis* MTCC 2413 was poured on agar plate and spread properly then allowed the culture to stable for about 20–30 min. After drying the wells were made and poured with 300 µl methanolic seed extracts of *S. cumini* Linn. Then plates were kept inside an incubator for overnight interval at 37 °C in an appropriate condition. Antimicrobial activity of methanolic seed extracts of *S. cumini* was determined by measuring zone of inhibitions using PBS as negative control. All the experiments were performed in triplicate.

### Minimum inhibitory concentration (MIC)

The minimum inhibitory concentration (MIC) of methanolic seed extract of *S. cumini* was determined by Clinical and Laboratory Standards Institute-(CLSI) recommended broth microdilution assay with few modifications. The methanolic seed extract was made in between 0.1 and 2.0 mg/ml. The microtiter plate were inoculated with 100 µl of bacterial culture and treated with prepared dilution of methanolic seed extract of *S. cumini* and overnight incubation was given at 37 °C. The absorbance was measured at 600 nm using microtiter plate reader to access the cell growth. The negative control was taken as LB broth bacteria suspension without any agent and blank as only medium and positive control as medium with agents. The MIC was calculated at lowest concentration at which it inhibits the growth of bacteria. All these experiments were performed in triplicate.

### Cell viability assessment by fluorescence microscopy

The *B. subtilis* cells were further stained with propidium iodide (PI), a nucleic acid staining dye and analysed by florescent microscope. The Bacterial cells were isolated from culture flask and centrifuged at 7000 rpm for 10 min at 4 °C to pellet down the cells. The cells were washed twice a time with phosphate buffer saline (PBS). The washed cells were fixed with 3.7% paraformaldehyde in PBS for 20 min at room temperature and then washed with cold PBS, and added propidium iodide. After adding Propidium iodide the cells were spread on the glass slide and covered by cover slip and observed by fluorescent microscope.

### Membrane permeabilization by flow cytometric analysis

To reveal the mechanism behind antibacterial activity of methanolic seed extract of *S. cumini*, and its ability to permeabilize bacterial membrane was done by PI uptake assay (Tyagi et al. 2015). Briefly *B. subtilis* was grown in LB agar media and treated with seed extract of *S. cumini* for 24, 48 and 72 h and kept in an incubator at 37 °C respectively. After every equal interval cells were harvested and washed twice with PBS, stained with Propidium iodide (PI) and incubated for 20 min in dark. The PI fluorescence was measured using flow cytometer. A total of 10,000 cells were taken for each flow cytometric analysis.

### Cells surface characterization by field emission scanning electron microscope

The field emission scanning electron microscope (FESEM) was performed to examine the morphological changes in *B. subtilis* MTCC 2413 strain after treatment with methanolic seed extract in varying time interval viz. 24, 48 and 72 h. Bacterial cells were centrifuged at 6000 rpm for 10 min to settle down the bacterial pellet. The bacterial pellets were washed twice in phosphate buffer saline (PBS) at 5000 rpm for 5 min. Washed bacterial pellet were fixed in 2.5% glutaraldehyde solution for 1 h. Fixed bacterial pellet were again washed with phosphate buffer saline (PBS) at 5000 rpm for 5 min. Washed bacterial pellet was dehydrated in series of 10, 20, 30, 40, 50, 60, 70, 80, 90 and 100% ethanol for 10 min at 37 °C. Bacterial pellet were put inside the incubator to completely dry at 65 °C for 2–3 days. The completely dried bacterial pellets were then coated with Palladium:Gold (80:20) before subjecting to field emission scanning electron microscope for observing the change in microbial morphology. The sample preparation for field emission scanning electron microscopy was done as per accordance of Teanpaisan et al. (2014) with minor modification.

### Cell apoptosis by DNA fragmentation

DNA fragmentation is considered as the biochemical hallmark of apoptosis (Halder et al. 2015). DNA fragmentation during apoptosis causes breakage of chromosomal DNA. Random degradation of DNA in bacterial cells will results in a diffuse smear upon electrophoresis of DNA. *B. subtilis* treated with methanolic extract of *S. cumini* were subjected to DNA fragmentation analysis according to procedure of Nagata (2000) with minor modification. *Bacillus subtilis* cells were incubated with lysis buffer containing SDS, Tris, EDTA for 1 h at room temperature. After lysis, supernatant was taken and DNA was extracted by chloroform:Isoamyl alcohol method and precipitated by ethanol. The precipitated DNA was dissolved in Tris–EDTA (TE) buffer and run on to 1.2% agarose gel stained with ethidium bromide for analysis.

### Molecular docking for potent target finding of *B. subtilis* MTCC 2413

#### File preparation

Structure files obtained from various database contains heteroatoms and sometimes even multiple copies of polypeptide chains, hence before docking ligand and macromolecule file must be processed to eliminate false positives and to get precise docking results.

#### Ligand preparation

3D structure of ligands was obtained from the pubchem database in sdf format which was converted into pdb format using free version of Discovery Studio tool. Structure was then modified using AutoDock Tool (ADT) version 4.2. Following modifications were made, addition of hydrogen atoms and addition of gestiger charges then file was saved in pdbqt format.

#### Macromolecule preparation

On the basis of cell wall cracking observed in bacterial SEM analysis macromolecule choice was restricted to only those which play role in peptidoglycan synthesis/breakdown. Four macromolecules from *B. subtilis* were chosen for docking. Macromolecule 3D structures with high structural resolution were downloaded from PDB server in pdb format. Obtained structure file were modified first to remove heteroatoms along with the water molecules secondly hydrogen atoms were added to all polar atoms to satisfy the valences and then gestiger charges were added to all the amino acid residues at last. After all modifications file was saved in pdbqt format. All structural modifications were done using ADT.

#### Docking

Docking analysis was performed by AutoDock ver. 4.2. Docking with AutoDock requires preparation of two kind of files, gpf (grid parameter file) and dpf file (docking parameter file). Grid parameter file defines the 3D search space for every atom type of a ligand by specifying points three dimensionally in the chosen grid, as well as their spacing. Docking parameter file on the other hand contains information about the docking algorithm to be used. Firstly grid parameter files were prepared for all the possible ligand macromolecule combinations hence a total of 44 gpf files were prepared. After making gpf, 44 docking parameter files were made using Lamarckian genetic algorithm. Using these gpf and dpf file glg (grid log file) and dlg (docking log file) were prepared. For every ligand-macromolecule docking a total of 10 different conformations were generated which later were analysed on the basis of their binding energy and force of attraction.

## Results

### Antimicrobial activity of methanolic seed extracts of *S. cumini* Linn. on *B. subtilis*

**Fig. 1:**
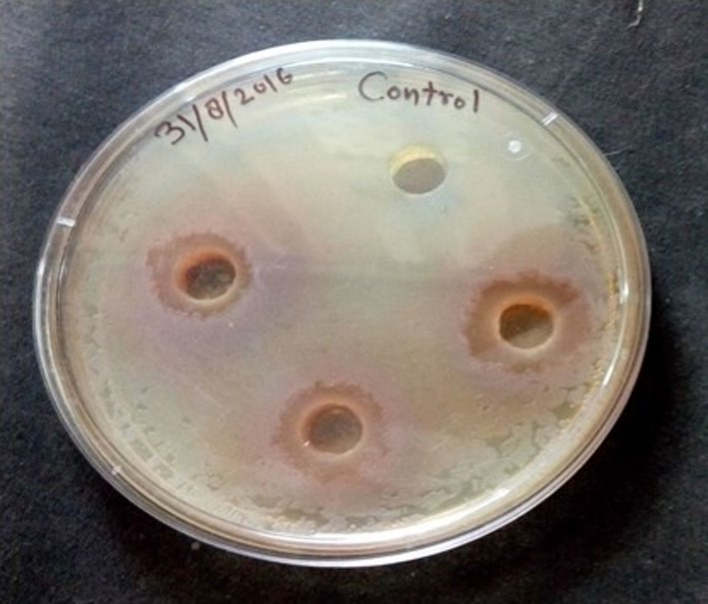
Antibacterial activity of *Syzygium cumini* methanolic seed extract against MTCC 2413 *Bacillus subtilis* at the concentration of 25 mg/ml

The methanolic seed extract of *S. cumini* was evaluated for the antimicrobial activity against the *B. subtilis* by agar well diffusion assay, the result is given in Fig. 1. The result showed that *B. subtilis* is susceptible against methanolic seed extract of *S. cumini*. The methanolic seed extract showed an inhibition zone of 20.03 mm. Here in this study we attempted to understand the antibacterial mechanism behind the methanolic extract of *S. cumini* on *B. subtilis*.

### Minimum inhibitory concentration (MIC)

The minimum inhibitory concentration was found to be 0.3 mg/ml with respect to control.

### Cell viability test

**Fig. 2:**
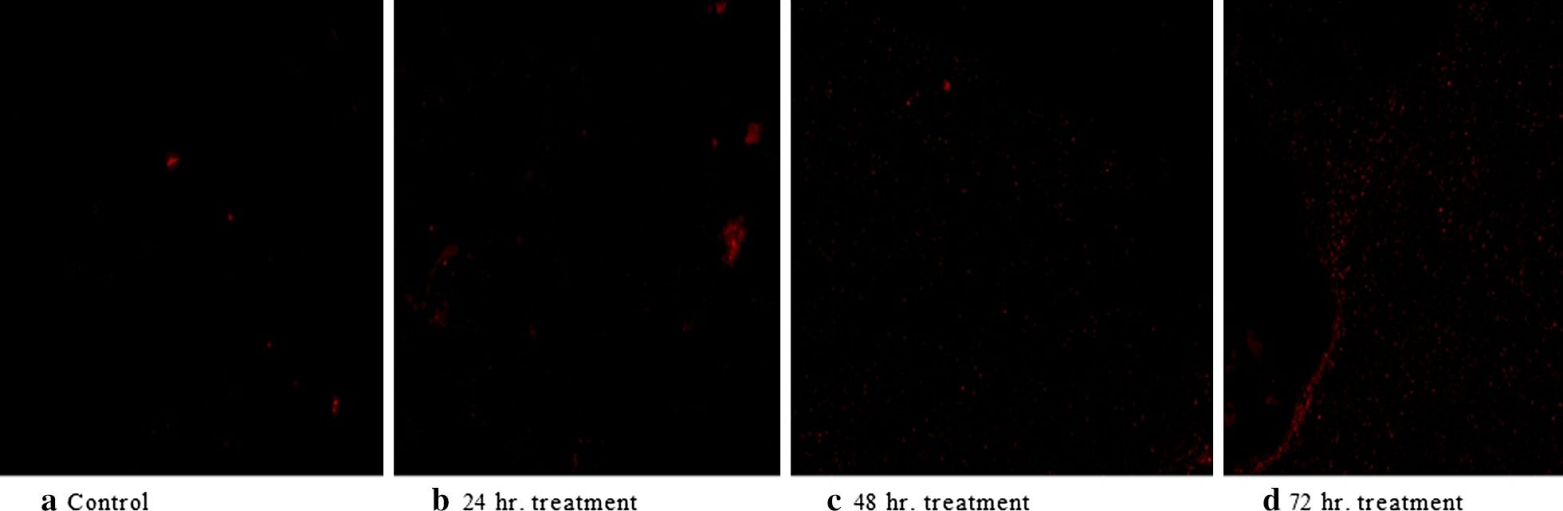
Fluorescence microscopy image of *Bacillus subtilis* stained with propidium iodide at varying period of time. **a** Control, **b** 24 h treatment, **c** 48 h treatment, **d** 72 h treatment

The *B. subtilis* MTCC2413 was subjected to methanolic seed extract of *S. cumini* for various time interval viz. 24, 48 and 72 h. The viability of *B. subtilis* was assessed by using fluorescent microscopy by staining cells with Propidium iodide (PI) at 20× magnifications. PI is commonly used as dead cell marker because live cell membrane excludes it and fluorescence is given only by dead cell whose membrane integrity is damaged. When *B. subtilis* were stained with PI red fluorescence appear as dead cell acquired the stain and appear as red colour spot when observed by fluorescence microscope (Fig. 2). Results shows the images of *B. subtilis* exposed to various time interval at the same concentration as observed by fluorescent microscope. It is clearly observed from the result that very few populations of bacterial cells were stained in control; however the population of stained bacterial cells increased with increasing time interval at the same concentration.

### Flow cytometric analysis for membrane permeabilization of *B. subtilis*

**Fig. 3:**
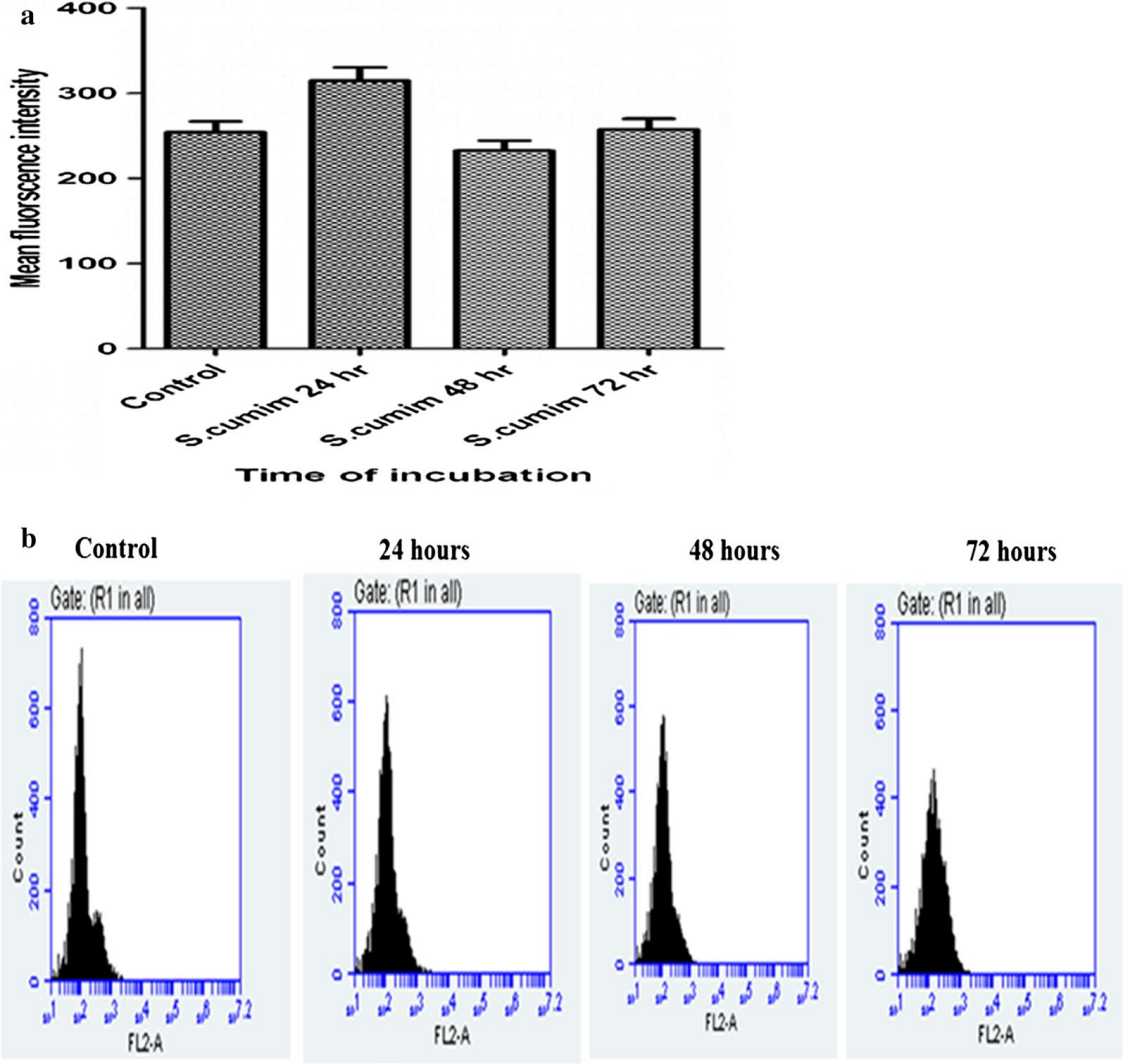
**a** Membrane permeabilization of *Bacillus subtilis* MTCC 2413 by *Syzygium cumini* methanolic seed extract, using propidium iodide (PI) through flow cytometer technique. A total of 10,000 cells were acquired for each flow cytometric analysis. **b** Mean fluorescence intensity of propidium iodide in *Bacillus subtilis* cells exposed to *Syzygium cumini* on same concentration at varying period of time. **b** Effect of *Syzygium cumini* methanolic seed extract on membrane permeabilization of *Bacillus subtilis* :Histograms of *Bacillus subtilis* bacterial cells treated with *Syzygium cumini* methanolic seed extract for 24, 48 and 72 h, then stained with PI and analysed by flow cytometer

The membrane permeabilization studies using PI were done on gram positive, *B. subtilis*. PI uptake in *B. subtilis* treated with methanolic seed extract of *S. cumini* is represented in Fig. 3a, b shows shift of mean fluorescence intensity in bar graph of 24, 48 and 72 h of treated *B. subtilis* cells as compared to control cells. The mean fluorescence intensity of untreated cells of *B. subtilis* were found to be 254.177 while 24, 48 and 72 h treated *B. subtilis* were found to be 314.716, 232.604 and 257.280.

The analysis revealed that seed extract has more potent membrane potential disruption activity which indicates its antimicrobial potential. The antimicrobial potential of methanolic seed extract of *S. cumini* could be restricted to inner membrane permeabilization at varying period of time.

### Field emission scanning electron microscope

The *B. subtilis* were treated for time interval of 24, 48 and 72 h respectively for the comparison of treated and untreated samples. The surface characterization of *B. subtilis* MTCC 2413 field emission scanning electron microscope results revealed that methanolic seed extracts of *S. cumini* induces a significant variation in the size of *B. subtilis* cells in comparison of control at 500 nm. After 24 h of treatment treated cells become smaller in comparison to control. Furthermore the SEM image also revealing that after 24 h of incubation with 25 mg/ml of extract, morphology of *B. subtilis* is getting changed with the adherence of its extract on the surface. The size of *B. subtilis* is shrinking from its original size as compared to control and there is occurring a crack in the bacterial cell wall indicating the initialization of cell wall rupturing.

**Fig. 4:**
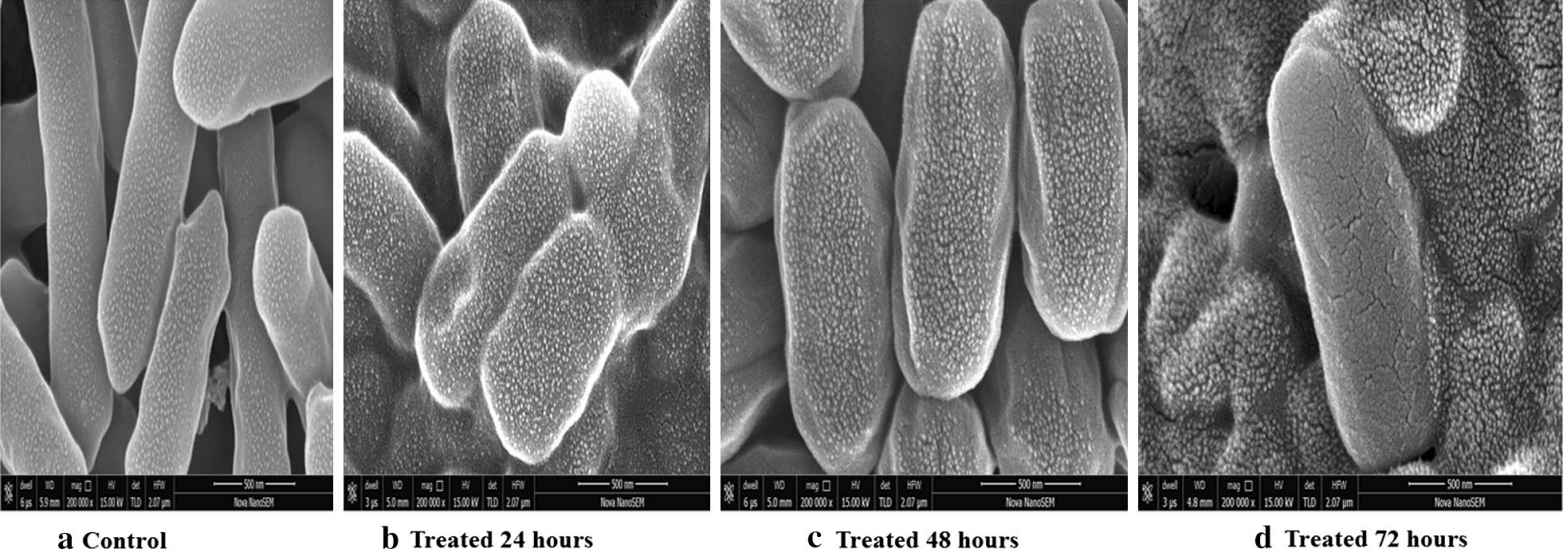
Field emission scanning electron microscope photographs of *Bacillus subtilis* MTCC2413 subjecting to 25 mg/ml at various time interval of 24, 48 and 72 h. The images were taken at 500 nm resolution. **a** Control, **b** treated 24 h, **c** treated 48 h, **d** treated 72 h

Similarly the size of *B. subtilis* is reduced further in ellipsoidal shape after treating it with methanolic extract of *S. cumini* for 48 h. After treatment of *S. cumini* extract for 48 h a clear cracking in the bacterial cell morphology is visible. Likewise at 72 h of treatment of methanolic seed extract of *S. cumini* on the bacterial cells, the bacterial cells lost their structural identity due to the cell wall degrading ability of extract. Here it is also clearly visible that cell wall breakage in the *B. subtilis* after 72 h treatment is more as compared to 24 and 48 h treatment respectively as shown in Fig. 4. The SEM image clearly indicates that methanolic seed extract exert a significant antibacterial effect on bacteria. It was also reported that the extract works in a time dependent manner Images at different time intervals indicate that as exposure of time increases bacterial death increases simultaneously.

### *Syzygium cumini* induced DNA fragmentation in *B. subtilis* MTCC 2413

**Fig. 5:**
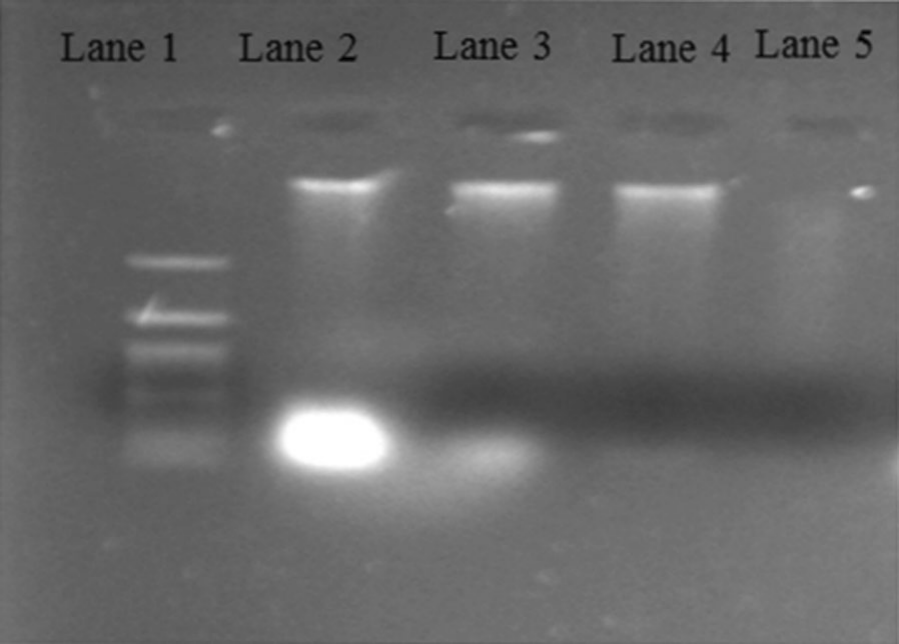
Apoptotic DNA fragmentation was observable in *Syzygium cumini* methanolic seed extract treated *Bacillus subtilis* MTCC2413 Lane 1—DNA ladder, lane 2—untreated genomic DNA (control) of *Bacillus subtilis* MTCC2413, lane 3—treated *Bacillus subtilis* MTCC 2413 DNA for 24 h, lane 4—treated *Bacillus subtilis* DNA MTCC 2413 DNA for 48 h, lane 5—treated *Bacillus subtilis* MTCC 2413 DNA for 72 h

To determine the DNA damage activity of methanolic seed extract of *S. cumini* in *B. subtilis* agarose gel electrophoresis was performed. The DNA fragmentation of methanolic seed extract of *S. cumini* in *B. subtilis* was in time dependent manner at constant concentration. No significant DNA fragmentation was observed after 24 h of treatment at constant concentration 25 mg/ml. While after treating *B. subtilis* at constant concentration for 48 h, a smear formation was observed indicating fragmentation initiation as shown in Fig. 5. Likewise on treating *B. subtilis* with constant concentration for 72 h, blurred visibility of DNA smear indicating DNA fragmentation. Furthermore methanolic seed extract of *S. cumini* is revealing that during DNA fragmentation RNA impurity at the bottom is getting degraded gradually with the passage of time. This indicates that methanolic seed extract has potent activity in the degradation of RNAs of *B. subtilis* and inhibition of bacterial pathogenic protein synthesis also.

### Molecular docking

In total 11 ligands (bioactive components in *S. cumini* L. methanolic extract) were selected for docking with 4 macromolecules chosen from *B. subtilis*. Detail of ligands and macromolecules are given in Table 1.

**Table 1:** Macromolecules chosen from *Bacillus subtilis*

S. no	PDB ID	Macromolecules from *Bacillus subtilis*
1	1W5D	DD-Carboxypeptidase
2	3MFD	β-Lactamase
3	4OX3	LD-Carboxypeptidase
4	5E2F	β-Lactamase class D

**Fig. 6:**
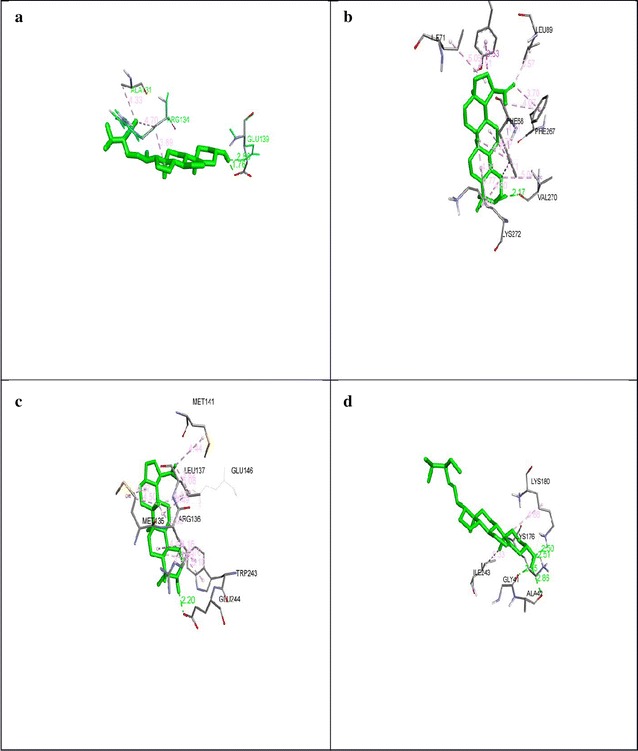
Various ligand/macromolecule docking interactions **a** interactions between betasitosterol and 5E2F involving two H-bonds with Glu139, **b** indicate lupeol interaction involving one H-bond with 4OX3, **c** represents interactions between lupeol and 1W5D with one H-bond, **d** represents stigmasterol and 3MFD forming four H-bonds

Docking analysis revealed that out of all the ligand chosen, lupeol showed highest binding energy for macromolecule with PDB id 1W5D and 4OX3 forming one H-bond each, on the other hand stigmasterol and betasitosterol showed the highest binding energy for macromolecule 3MFD and 5E2F respectively forming 4 and 2 H-bonds as shown in Fig. 6. These findings when combined with SEM analysis indicate that lupeol, stigmasterol and betasitosterol somehow effect the functioning of the chosen macromolecules in bacteria effecting their functioning leading to change in peptidoglycan structure. Ligand/macromolecule complex binding energy along with the bonding involved is given in Table 2

**Table 2:** Compounds from *Syzygium cumini* having high affinity for macromolecules from *Bacillus subtilis*

S. no	Compound	Macromolecules from *B. subtilis*	Binding energy	Hydrogen bonds	Bond length (Å)
1	Lupeol	1W5D	− 8.48	Glu244	2.20
2	Stigmasterol	3MFD	− 9.34	Lys180	2.50
				Lys176	2.51
				Gly41	2.05
				Ala42	2.86
3	Lupeol	4OX3	− 8.37	Val270	2.17
4	Betasitosterol	5E2F	− 6.42	GLU139	1.76
				GLU139	2.88

## Discussion

In comparison to the above results following data is presented as discussed below stepwise. The antibacterial activity of *S. cumini* is also reported in the fruit and leaf of it in methanol, chloroform and petroleum ether over the bacterial strain of *Raoultella planticola, Pseudomonas aeruginosa*, *B. subtilis* and *Agrobacterium tumifaciens* and the maximum zone of inhibition was observed in *R. plantikola* (25 mm) followed by *B. subtilis* (19 mm), *P. aeruginosa* (14 mm) and *A. tumifaciani* (12 mm) in the methanolic extract of *S. cumini* leaf (Pareek et al. 2015). The methanolic fruit extract of *S. cumini* also showed potent antibacterial activity against these bacteria in decreasing order of activity *R. plantikola* (21 mm) < *B. subtilis* (18 mm) < *P. aeruginosa* (13 mm) < *A. tumifaciani* (10 mm). The chloroform and petroleum ether extract of *S. cumini* showed potent antibacterial property against them likewise. The antimicrobial activity of *S. cumini* hydro alcoholic leaf extract was also evaluated against mutli resistant strain of *P. aeruginosa*, *Klebsiella pneumonia* and *Staphylococcus aureus* and showed an inhibition zone of 10, 0.0 and 9.0 mm respectively (Oliveira et al. 2007). *Syzygium cumini* methanolic and aqueous leaf extracts were tested against some gram positive (*B. subtilis* and *S. aureus*) and gram negative bacteria (*Salmonella enteritidis*, *Salmonella typhi A*, *Salmonella para typhi A, Salmonella para typhi B, Pseudomonas aeruginosa, E. coli*) and concluded that methanolic leaf extract has more better antimicrobial activity as compared to aqueous extracts (Gowri and Vasantha 2010).

Evaluating the MIC of *S. cumini* also showed minimum inhibitory concentration of hydroalcoholic leaf extract of *S. cumini* against multi resistant strain of *Pseudomonas aeruginosa* was found to be 80 µmg/ml. The MIC of methanolic fruit extract of *S. cumini* was found to be 0.18 mg/ml compared to ethyl acetate (0.48 mg/ml) and dichloromethane extract (0.52 mg/ml) of *S. cumini* fruit against *Enterococcus faecalis* (Priya et al. 2013).

Propidium iodide intercalates into double standard nucleic acid. It is excluded by live cells but can penetrate inside the disrupted membrane of dead cells. It is a routine parameter used for determining the apoptosis of disrupted membrane of dead cells.

Bacterial isolates treated with various concentration of chromium showed that the population of stained cells of *B. subtilis* increased with increasing concentrations of chromium as compared to control (Upadhyay et al. 2017). *Brucea javanica* oil induced apoptosis in T 24 cells as analysed by PI (Lou et al. 2010). Propidium iodide staining revealed that an increase in apoptosis was noticed in imatinib exposure in time and dose dependent manner on C6 glioma cells of rat (Ling et al. 2010). On the basis of these results it showed that PI staining in the present study was highly validated.

It is believed that the action of many antimicrobial agents results in the formation of pores in bacterial membrane and stimulates leakage of cellular content (Yenugu et al. 2006). The ability of methanolic seed extract of *S. cumini* to cause cellular leakage indicated that they cause pores in the bacterial membrane. The membrane damaging activity of methanolic seed extract of *S. cumini* is mainly accounted in the phenols and flavonoids having detergent like characteristics. The antibacterial mechanism of alcoholic extracts of *Hemidesmus indicus* (L.) R. Br. Ex Schult, *Leucas aspera* (Wild.), *Plumbago zeylanica* L., and *Tridax procumbens* (L.) R. Br. ex Schult also have shown their antimicrobial action by blebbing and leakage of cellular contents (Saritha et al. 2015). The ethanolic extract of *Mentha arvensis* also induce cellular damage which increased with an increase in concentration (Zhang et al. 2015). Scanning electron microscopic analysis of *S. mutans* and *A. actinomycetemcomitans* has revealed that cells after treatment of aqueous extract of *A. lakoocha* lost their original shape having an irregular, distorted cell wall structure (Teanpaisan et al. 2014). The field emission scanning electron microscope has revealed that methanolic seed extract of *S. cumini* has same antimicrobial mechanism as compared to other plant extract.

The DNA fragmentation is considered as a major key feature of programmed cell death that occurs at certain stages of necrosis. The formation of clear DNA smear in case of treated sample indicates that it shows programmed cell death after treating *B. subtilis* cells with constant concentration at 24, 48 and 72 h as shown in Fig. 5. Pathogenic bacteria like *E. coli, S. aureus* and *K. pneumonia* on treatment with *Caesalpinia coriaria* glycosides and flavonoids for 24 h showed a smear of fragmented DNA (Anandhi et al. 2014). Similarly the ethanolic leaf extract of *S. cumini* after treating *V. cholerae* showed fragmented DNA after 3 h of treatment (Ahsan et al. 2012).

*Leucas aspera, Plumbago zeylanica, Hemidesmus indicus* ethanolic extract has shown potential of inner membrane permeabilization in *E. coli* (Saritha et al. 2015). The membrane potential disruption potential of some plants is reported such as *Gracilaria tenuistipitata* methanolic extract had reduced the mitochondrial membrane potential in MEGT-treated Ca9-22 cancer cells (Yeh et al. 2012). *Annona muricata* extract induced apoptosis in human cancer cell through disruption of mitochondrial membrane potential (Pieme et al. 2014).

Study of protein–protein or protein-receptor interactions through in silico analysis has been proved to be an efficient tool for drug designing. Similar study conducted that revealed benzofuran having high affinity for active site of COX-2 receptor showing it to be a good anti-inflammatory compound (Yadav et al. 2014). Molecular docking of several compounds was performed by Valasani et al. (2014) for a potential inhibitor for cyclophilin D, a crucial mitochondrial membrane protein. Molecular docking of methanolic seed extract of *S*. *cumini* with specific ligands revealed that these compounds could be used for antimicrobial agents for inhibition of *B. subtilis* growth. These results have provided an insight behind antimicrobial activity on the basis of binding energy. The binding energy of lupeol showed highest binding energy for macromolecule with PDB id 1W5D and 4OX3 forming one hydrogen bond each, on the other hand stigmasterol showed the highest binding energy for macromolecule 3MFD and 5E2F forming 4 hydrogen bonds and alkyl bonds respectively.

In conclusion, we reported that methanolic seed extract of *S. cumini* has potent antibacterial activity against *B. subtilis*. Although the mechanism behind the action of methanolic seed extract involves disrupting the bacterial cell wall, shrinkage in size, leaking of cellular contents. The antibacterial potential of methanolic seed extract of *S. cumini* can be exploited perfectly to treat infections in place of commonly used antibiotics in our day to day life. Natural replacement of chemical food preservative with herbal antimicrobial agents may provide a new and safe way to preserve the food from bacterial contamination. Futhermore, the antibacterial action of methanolic seed extract could be improved by using it in combination with natural herbal dietary phytochemicals such as phytic acid, resveratrol etc. which may increase the shelf life and control the food deterioration.

## Abbreviations

PI: Propidium iodide
MTCC: Microbial Type Culture Collection
FE-SEM: Field Emission Scanning Electron Microscope
MIC: Minimum Inhibitory Concentration
ADT: AutoDock Tool
EDTA: ethylene diamine tetra acetic acid
SDS: sodium dodecyl sulphate
